# Supplementary material for: Comprehensive Annotation and Functional Exploration of MicroRNAs in Lettuce
Source: Front Plant Sci. 2021 Dec 24;12:781836. doi: 10.3389/fpls.2021.781836 (PMC8739914; doi:10.3389/fpls.2021.781836)
Supplement: Supplementary file 2 [file Data_Sheet_8.ZIP › GSM2230749.plot/Lsa-miRN1690_Lsat_1_v5_gn_6_67720.1_1410_TPlot.pdf]

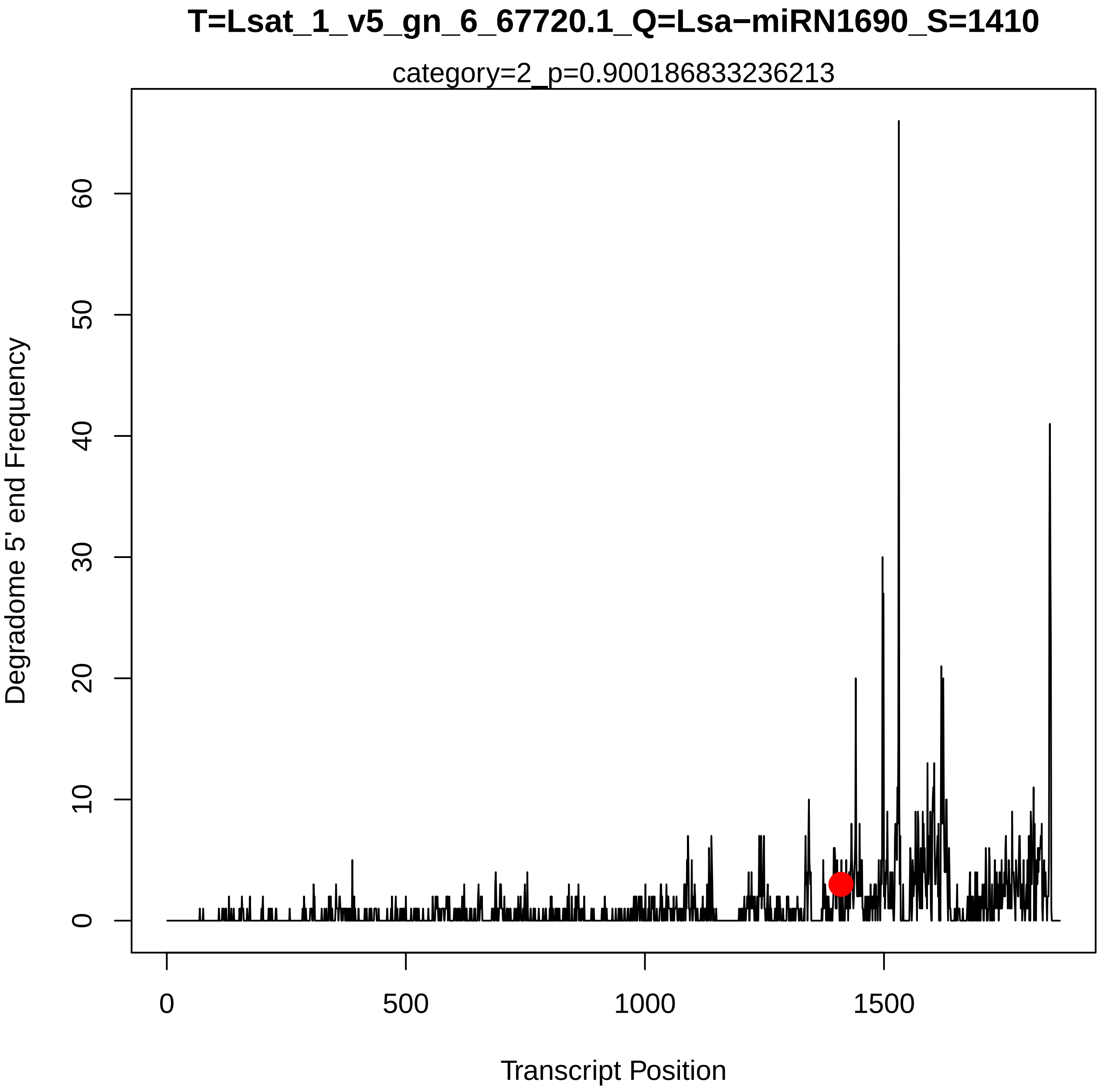
T=Lsat_1_v5_gn_6_67720.1_Q=Lsa−miRN1690_S=1410
category=2_p=0.900186833236213
Degradome 5' end Frequency
Transcript Position
0
10
20
30
40
50
60
0
500
1000
1500
